# SARS-CoV-2 Omicron variant virus isolates are highly sensitive to interferon treatment

**DOI:** 10.1038/s41421-022-00408-z

**Published:** 2022-05-10

**Authors:** Denisa Bojkova, Tamara Rothenburger, Sandra Ciesek, Mark N. Wass, Martin Michaelis, Jindrich Cinatl

**Affiliations:** 1 Institute for Medical Virology, University Hospital, Goethe University, Frankfurt am Main, Germany; 2 German Center for Infection Research, DZIF, External partner site, Frankfurt am Main, Germany; 3 Fraunhofer Institute for Molecular Biology and Applied Ecology (IME), Branch Translational Medicine und Pharmacology, Frankfurt am Main, Germany; 4 School of Biosciences, University of Kent, Canterbury, UK; 5 Dr. Petra Joh-Forschungshaus, Frankfurt am Main, Germany

**Keywords:** Innate immunity, Mechanisms of disease

Dear Editor,

The SARS-CoV-2 Omicron variant (B.1.1.529) causes less severe disease than previous SARS-CoV-2 variants, although immune protection provided by vaccinations and previous infections is reduced against Omicron compared to previous variants^1^. In agreement, evidence is emerging that Omicron is inherently less pathogenic than previous SARS-CoV-2 variants. Omicron variant viruses cause less severe disease in animal studies^2^ and appear to display a lower capacity than other variants to replicate in the lower respiratory tract^2^. Additionally, initial clinical data indicated that the Omicron variant causes less severe disease than previous SARS-CoV-2 variants in unvaccinated individuals^1^.

We have most recently shown that Omicron variant viruses are less effective at antagonizing the host cell interferon response than Delta variant viruses^3^, which provides a mechanistic explanation for the reduced clinical severity of Omicron disease in individuals without pre-existing adaptive immunity^1^. Omicron virus replication was attenuated relative to Delta virus in interferon-competent Caco-2 and Calu-3 cells, but not in interferon-deficient Vero cells, and Omicron viruses caused enhanced interferon promoter activity compared to Delta viruses^3^. Additionally, depletion of the pattern recognition receptor MDA5, which plays a critical role in SARS-CoV-2 detection and interferon response initiation^4^, resulted in increased Omicron virus replication in interferon-competent cells^3^.

The exact molecular reasons for the alleviated interferon response antagonism by Omicron viruses remain to be elucidated. Notably, the Omicron and Delta virus isolates that we investigated (see Supplementary Information) display sequence variants in the viral interferon antagonists nsp3, nsp12, nsp13, nsp14, the membrane (M) protein, the nucleocapsid protein, and ORF3a^5^ (Supplementary Table S1), which may be of relevance.

Here we further show that two SARS-CoV-2 Omicron isolates (Omicron 1, Omicron 2) replicate to lower titers (Fig. 1a) and induce elevated STAT1 phosphorylation (Fig. 1b), a key event during interferon signaling, compared to a Delta isolate (B.1.167.2) in Caco-2 and Calu-3 cells^3^ (see Supplementary Information).

**Fig. 1:**
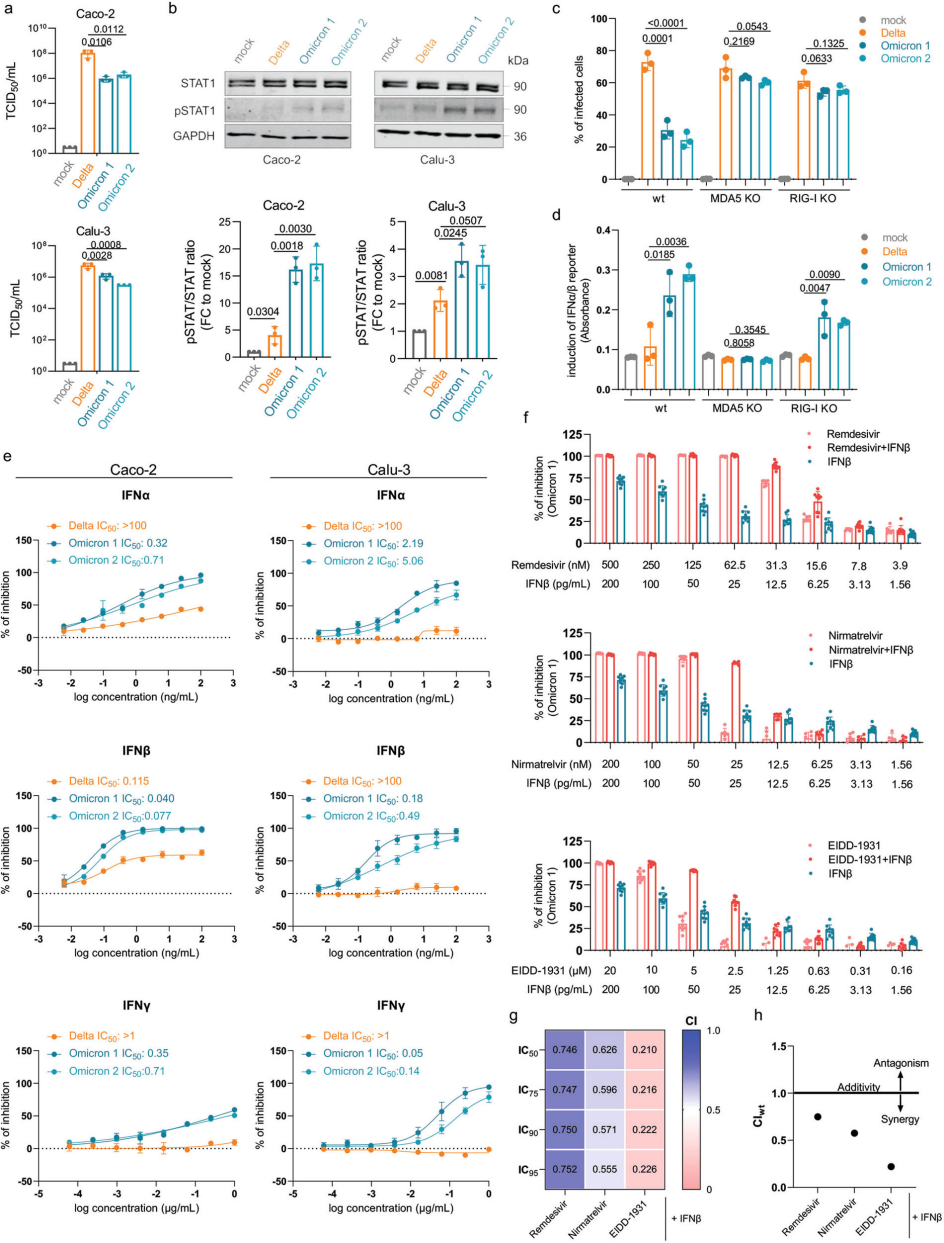
IFN signaling and therapy during infection with novel SARS-CoV-2 variant Omicron. **a** Caco-2 and Calu-3 cells were infected with SARS-CoV-2 variant Delta (GenBank ID: MZ315141), Omicron 1 (GenBank ID: OL800702), and Omicron 2 (GenBank ID: OL800703) at an MOI of 1. The infectious titer was determined 24 h post infection. Graphs represent means ± SD of three biological replicates. Statistically significant differences were identified by one-way ANOVA and subsequent Dunnett’s test. **b** Immunoblot analysis of total and phosphorylated STAT1 in SARS-CoV-2-infected Caco-2 (left panel) and Calu-3 (right panel) cells 24 h post infection. The protein levels were quantified by ImageJ. Graphs represent means ± SD of three biological replicates. *P* values were calculated using Student’s *t* test. **c** A549-ACE2/TMPRSS2 MDA5/RIG-I-WT (wt), A549-ACE2/TMPRSS2 MDA5 KO (MDA5 KO), and A549-ACE2/TMPRSS2 RIG-I KO (RIG-I KO) cells were infected with Delta, Omicron 1, and Omicron 2 variants at an MOI of 0.01 for 72 h. The number of infected cells was determined by immunofluorescence staining. Graphs represent data of four biological replicates. Statistically significant differences were identified by one-way ANOVA and subsequent Dunnett’s test. **d** Production of IFNα/β was measured by incubating supernatants from wt, MDA5-KO, and RIG-I KO cells infected with SARS-CoV-2 variants at an MOI of 0.01 for 48 h using HEK-Blue IFNα/β reporter cells. Graphs displays means ± SD of three biological replicates. Statistically significant differences were identified by one-way ANOVA and subsequent Dunnett’s test. **e** Dose–response curves of IFNα, IFNβ, and IFNγ were determined in Caco-2 and Calu-3 cells. All IFNs were added to confluent monolayers and subsequently infected with viral variants at an MOI of 0.01. The inhibition rate was evaluated 24 h (Caco-2) and 48 h (Calu-3) post infection by staining for the spike protein. Graphs depict means ± SD of three biological replicates. **f** Antiviral effect of IFNβ in combination with nirmatrelvir, remdesivir, or EIDD-1931 in Caco-2 cells. Statistically significant differences were identified by one-way ANOVA and subsequent Dunnett’s test. **g** Heatmap of combination indexes (CIs) for IC_50_, IC_75_, IC_90_, and IC_95_. **h** Dots depict weighted average (CI_wt_) calculated according to the formula: CI_wt_ = (CI_50_ + 2CI_75_ + 3CI_90_ + 4CI_95_)/10. CI_wt_ < 1 synergism, CI_wt_ = 1 additive effects, CI_wt_ > 1 antagonism.

In A549 cells transduced with ACE2 (SARS-CoV-2 receptor) and TMPRSS2 (mediates SARS-CoV-2 cell entry by cleaving and activating the viral spike protein), the Omicron viruses also displayed alleviated infection capacity compared to the Delta virus (Fig. 1c). This difference largely disappeared upon depletion of either of the pattern recognition receptors MDA5 and RIG-I, both of which mediate the host cell interferon response in virus-infected cells^6^. However, when we compared interferon activity in the supernatants of SARS-CoV-2-infected cells in a HEK-Blue IFNα/β reporter cell assay, the supernatants of Omicron virus-infected RIG-I-knock out cells induced higher interferon promoter activation than the supernatants of Omicron virus-infected MDA5-knock out cells (Fig. 1d). This is in agreement with previous data showing that MDA5 is primarily responsible for virus recognition and the induction of an interferon response in SARS-CoV-2-infected cells^4,6,7^.

Taken together, these findings further confirm that Omicron viruses are less effective than Delta viruses in antagonizing the host cell interferon response^3^ and that MDA5 is a major player in SARS-CoV-2 recognition^4,6^. Accordingly, elevated MDA5 levels were detected in the upper airways of SARS-CoV-2-infected individuals with mild or asymptomatic disease^8^. Since Delta has been found to display a similar level of interferon antagonism and sensitivity as previous SARS-CoV-2 variants^9,10^, the reduced interferon antagonism appears to be unique to Omicron.

Most notably, treatment with interferon-α, interferon-β, and interferon-γ revealed that the weaker interferon antagonism by Omicron virus isolates translates into a profoundly increased Omicron sensitivity to interferon treatment (Fig. 1e). Further experiments showed that antiviral interferon-β effects were further increased in combination with nirmatrelvir (the antivirally active agent in paxlovid), remdesivir, and EIDD-1931 (the active metabolite of molnupiravir) (Fig. 1f). Combination experiments using the Chou–Talalay approach^11^ indicated moderate synergism of interferon-β with remdesivir, synergism with nirmatrelvir, and strong synergism with EIDD-1931 (Fig. 1g, h). So far, clinical studies reported mixed outcomes in COVID-19 patients treated with different interferons^12–15^. Given the newly discovered substantially increased interferon sensitivity of Omicron viruses, interferons represent a promising option for the treatment of Omicron patients.

In conclusion, we present further evidence that reduced interferon-antagonizing activity explains at least in part why Omicron variant infections are inherently less severe than infections with other SARS-CoV-2 variants. Sequence variations in the SARS-CoV-2 interferon antagonists nsp3, nsp12, nsp13, nsp14, M protein, the nucleocapsid protein, and/or ORF3a may contribute to these differences. Most importantly, this study shows that Omicron variant viruses display enhanced sensitivity to interferon treatment, which makes interferons promising therapeutic candidates for Omicron patients, in particular in combination with other antiviral agents.

## Supplementary information

Supplementary Information
